# The Underlying Mechanisms for Olanzapine-induced Hypertriglyceridemia

**DOI:** 10.4021/jocmr802w

**Published:** 2012-05-15

**Authors:** Hiroki Adachi, Hidekatsu Yanai, Yuji Hirowatari

**Affiliations:** a Department of Internal Medicine, National Center for Global Health and Medicine, Kohnodai Hospital, Chiba 272-8516, Japan; b Clinical Research Center, National Center for Global Health and Medicine, Kohnodai Hospital, Chiba 272-8516, Japan; c Bioscience Division, Tosoh Corporation, Kanagawa, Japan; d Hiroki Adachi and Hidekatsu Yanai contributed equally to this study.

**Keywords:** Adiponectin, Hypertriglyceridemia, Inflammation, Olanzapine

## Abstract

Olanzapine is an efficacious antipsychotic drug often used in the treatment for schizophrenia or bipolar disorder, however, sometimes induces metabolic disorders. We will introduce a patient with bipolar disorder, who has been treated by olanzapine and showed severe hypertriglyceridemia. As a result of measurements of parameters associated with lipid metabolism, very-low density lipoprotein was most important lipoprotein for olanzapin-induced hypertriglyceridemia. The cessation of olanzapine significantly decreased high-sensitivity C-reactive protein and increased adiponectin, proposing that inflammation and reduced adiponectin level may be associated with olanzapin-induced hypertriglyceridemia.

## Introduction

Olanzapine is an efficacious antipsychotic drug often used in the treatment for schizophrenia or bipolar disorder, however, sometimes induces metabolic disorders such as obesity and hypertriglyceridemia [1, 2]. We will introduce a patient with bipolar disorder, who has been treated by olanzapine and showed severe hypertriglyceridemia. Here, we will show the changes in levels of triglyceride (TG), TG-rich lipoproteins, lipoprotein lipase (LPL), adiponectin, and high-sensitivity C-reactive protein (hs-CRP) at 1 and 2 months after the cessation of olanzapine, which may advance the understanding of the underlying mechanisms for olanzapine-induced hypertriglyceridemia.

## Case Report

**Figure 1:**
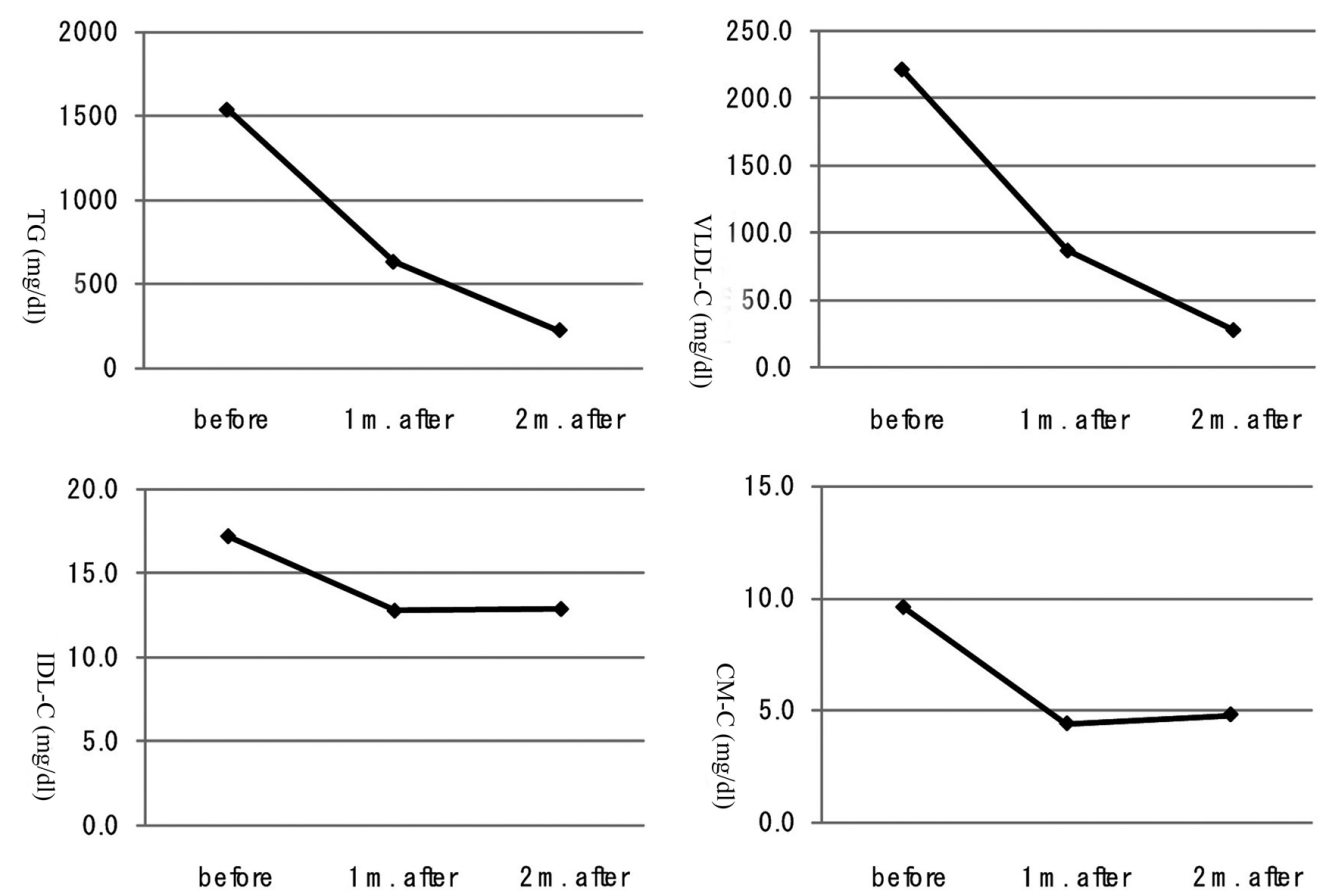
Changes in triglyceride (TG), very low-density lipoprotein-cholesterol (VLDL-C), intermediate-density lipoprotein-cholesterol (IDL-C) and chylomicron-cholesterol (CM-C) at 1 month (m.) and 2 months (m.) after the cessation of olanzapine.

**Figure 2:**
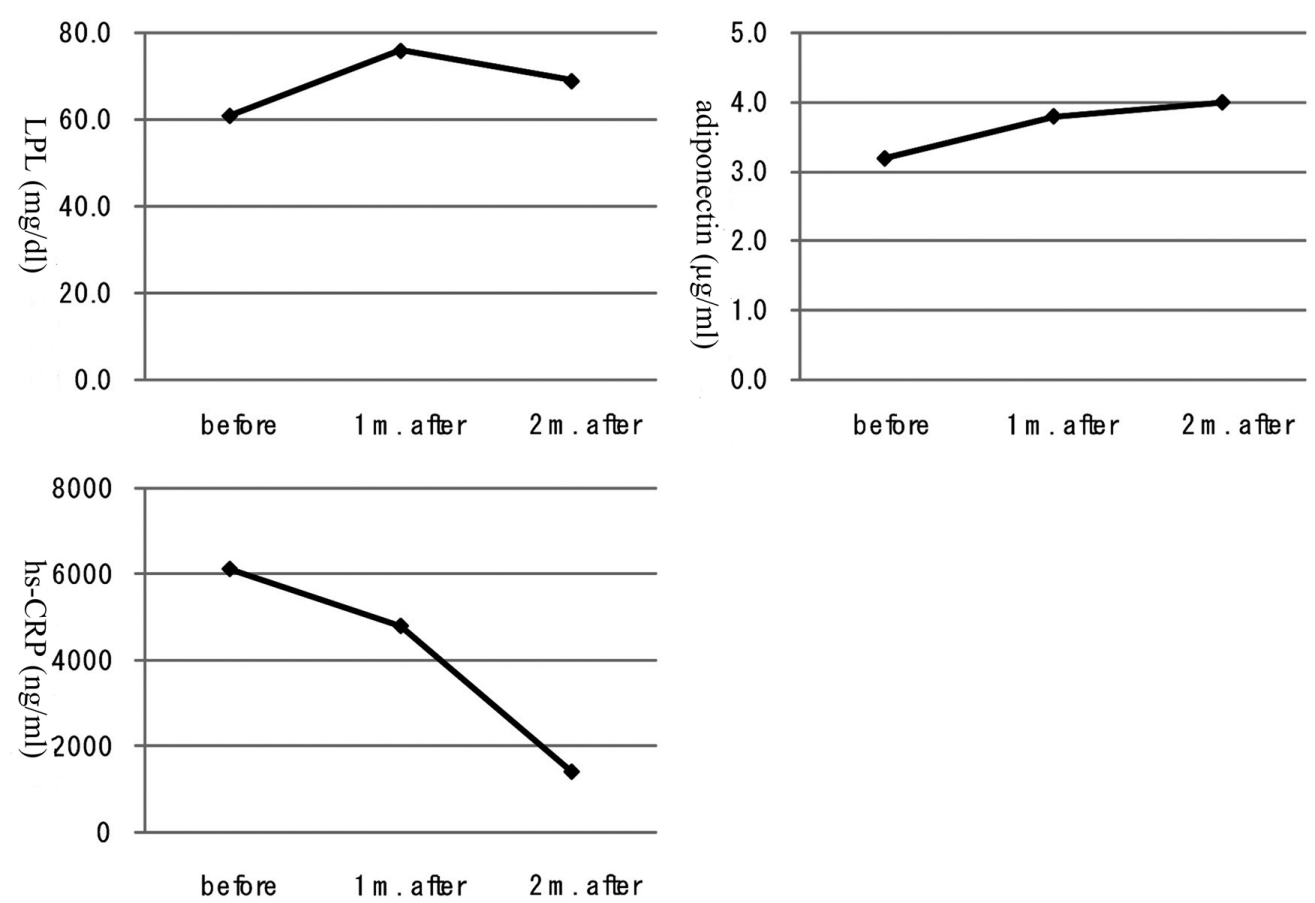
Changes in lipoprotein lipase (LPL), adiponectin and high-sensitivity C-reactive protein (hs-CRP) at 1 month (m.) and 2 months (m.) after the cessation of olanzapine.

A 40-year-old man was referred to our department due to severe hypertriglyceridemia (TG: 1,598 mg/dl) in August 2010. His body weight was 67 kg and height 170 cm (BMI: 23.2 kg/m^2^). At the age of 23 he has been diagnosed as bipolar disorder, and the treatment using olanzapine started in March 2008. He has been treated by using levomepromazine (10 mg/day), lithium carbonate (800 mg/day), flunitrazepam (2 mg/day) and olanzapine (10 mg/day). After the cessation of olanzapine, he was treated by levomepromazine (10 mg/day), lithium carbonate (800 mg/day), flunitrazepam (2 mg/day) and quetiapine fumarate (50 mg/day). Cessation of taking olanzapine did not change his body, however, promptly decreased serum TG level (Fig. 1). To understand which TG-rich lipoprotein is important for olanzapine-induced hypertriglyceridemia, we measured each lipoprotein fraction by the newly developed anion-exchange high-performance liquid chromatography [3]. Serum very low-density lipoprotein cholesterol (VLDL-C) level was remarkably high during the olanzapine use, and was also promptly decreased after the cessation of olanzapine, and the decrease of VLDL-C almost paralleled the decrease of TG (Fig. 1). Serum levels of other TG-rich lipoproteins, intermediate-density lipoprotein (IDL)-C and chylomicron (CM)-C, decreased at one month after the cessation, however, again increased slightly at two months after the cessation (Fig. 1). Serum LPL levels increased at one month after the cessation, however, again decreased slightly at two months after the cessation (Fig. 2). Serum adiponectin level was constantly increased, and hs-CRP level was constantly and significantly decreased after the cessation of olanzapine (Fig. 2).

## Discussion

Present study suggested that VLDL is most important TG-rich lipoprotein for olanzapine-induced hypertriglyceridemia. The association of defective LPL activity to an increase in VLDL may be limited, which is supported by the small increase of LPL and the small decrease of IDL-C and CM-C after the cessation of olanzapine. The cessation of olanzapine leads to a significant decrease in hs-CRP and increase in adiponectin, proposing that the main underlying mechanism for olanzapine-mediated increase in VLDL may be olanzapine-induced inflammation and reduced adiponectin. Briefly, olanzapine may induce inflammation and reduce adiponectin, leading to activation of hormone-sensitive lipase which hydrolyzes TG to free fatty acids (FFA) [4]. Increased circulating FFA may enter the liver, resulting in hepatic overproduction of VLDL.

In conclusion, our study demonstrated that VLDL is most important TG-rich lipoprotein for olanzapine-induced hypertriglyceridemia. The cessation of olanzapine leads to a significant decrease in hs-CRP and increase in adiponectin, proposing that inflammation and reduced adiponectin level may be associated with olanzapine-induced hypertriglyceridemia.
